# Early Postnatal Comprehensive Biomarkers Cannot Identify Extremely Preterm Infants at Risk of Developing Necrotizing Enterocolitis

**DOI:** 10.3389/fped.2021.755437

**Published:** 2021-10-22

**Authors:** Alice Hoffsten, Laszlo Markasz, Helene Engstrand Lilja, Karl Wilhelm Olsson, Richard Sindelar

**Affiliations:** ^1^Department of Women's and Children's Health, Uppsala University, Uppsala, Sweden; ^2^Neonatal Intensive Care Unit, University Children's Hospital, Uppsala, Sweden; ^3^Section of Pediatric Surgery, University Children's Hospital, Uppsala, Sweden; ^4^Department of Surgical Sciences, Uppsala University, Uppsala, Sweden

**Keywords:** necrotizing enterocolitis, biomarker, preterm infant, cluster analysis, serum

## Abstract

**Background:** Necrotizing enterocolitis (NEC) is a fatal disease where current diagnostic tools are insufficient for preventing NEC. Early predictive biomarkers could be beneficial in identifying infants at high risk of developing NEC.

**Objective:** To explore early biomarkers for predicting NEC in extremely preterm infants (EPIs).

**Methods:** Blood samples were collected on day 2 (median 1.7; range 1.5–2.0) from 40 EPI (median 25 gestational weeks; range 22–27): 11 developed NEC and 29 did not (controls). In each infant, 189 inflammatory, oncological, and vascular proteomic biomarkers were quantified through Proximity Extension Assay. Biomarker expression and clinical data were compared between the NEC group and Controls. Based on biomarker differences, controls were sorted automatically into three subgroups (1, 2, and 3) by a two-dimensional hierarchical clustering analysis.

**Results:** None of the biomarkers differed in expression between all controls and the NEC group. Two biomarkers were higher in Control 1, and 16 biomarkers were lower in Control group 2 compared with the NEC group. No biomarker distinguished Control 3 from the NEC group. Perinatal data were similar in the whole population.

**Conclusions:** Early postnatal comprehensive biomarkers do not identify EPIs at risk of developing NEC in our study. Future studies of predictors of NEC should include sequential analysis of comprehensive proteomic markers in large cohorts.

## Introduction

Even though mortality rates among preterm infants have halved during the past two decades (1), the aim to reduce it further continues. Recent studies (2–5) have found an upsurge of necrotizing enterocolitis (NEC) as cause of death. The increase of mortality from NEC can be due to successful care of other early illnesses, allowing the preterm infants to survive long enough to be susceptible to NEC (6, 7).

Since the first description of NEC (8), decades have been devoted to understand its pathogenesis and etiology. The recent knowledge on NEC suggests that mechanisms are multifactorial with both prenatal and postnatal factors. Current consensus proposes that NEC is an inflammatory disease, where injuries to the intestinal wall barrier lead to bacterial invasion and necrosis (6). The immaturity of the preterm gut barrier and the developing, over-reactive immune system enhance the response and destruction (9). Exaggerated mucosal inflammation and necrosis may be amplified by abnormal microcirculation (10). Postnatally, diet (11, 12) and epidermal growth factors (13) will affect intestinal maturation and can thus have an impact on the susceptibility of NEC. Furthermore, low birth weight (BW), being small for gestational age (SGA), anemia, and increased immaturity are all recognized risk factors of NEC (14, 15).

Besides substantial mortality, NEC is associated with longer hospital stays (16), impaired neurodevelopment (17), and morbidity due to short bowel syndrome following lifesaving surgical interventions (18).

Diagnosis of NEC is based on the modified Bell's staging criteria, consisting of radiographic, clinical, and laboratory findings (19, 20). Early diagnosis is more challenging in more immature infants, due to non-specific clinical and radiographic signs (21). Desolately, it is also in the most premature newborns that the mortality and incidence of NEC are the largest (22). It is therefore of great interest to find a more reliable and earlier diagnosis of NEC in these individuals. This would enable earlier intervention and thus reduce progression, morbidity, and mortality of NEC. A precise diagnosis will also decrease over-treatment (23).

Plasma proteins have been proven useful in identifying diseases in extremely preterm newborns, for instance, in bronchopulmonary disease (BPD), patent ductus arteriosus (PDA), and retinopathy of prematurity (ROP) (23–26). Many attempts have been made to identify biomarkers in serum, stool, and urine for early diagnosis of NEC, but the clinical relevance of these findings still remains low. Individual inflammatory biomarkers are usually non-specific, reflect general inflammation rather than specifically NEC, and are detected at later stages of NEC (24–26). Thus, they do not facilitate an earlier diagnosis. Some biomarkers require the infant to pass stool, which is not always possible in advanced NEC (25, 27). Inter-individual and intra-individual variations have also been obstacles to exploring useful biomarkers (28). Other difficulties for finding predictive biomarkers in NEC could be that the correlation between biomarkers and disease is not as linear, for instance, between vascular endothelial growth factor (VEGF) and ROP, and that NEC probably is multifactorial. The individuals at risk of developing NEC may also display heterogeneity and have different risks. Therefore, exploring combinations of comprehensive biomarkers in infants at the highest risk of developing NEC may be useful.

The aim was to prospectively study early comprehensive biomarkers in serum from extremely preterm infants who might or might not develop NEC. Biomarker patterns in healthy infants are compared with patterns in those who later developed NEC. Besides finding useful potential early biomarkers to improve current diagnostic tools in this high-risk population, this could also yield valuable information about the pathophysiology of NEC.

## Methods and Materials

### Study Setting and Participants

**Figure 1:**
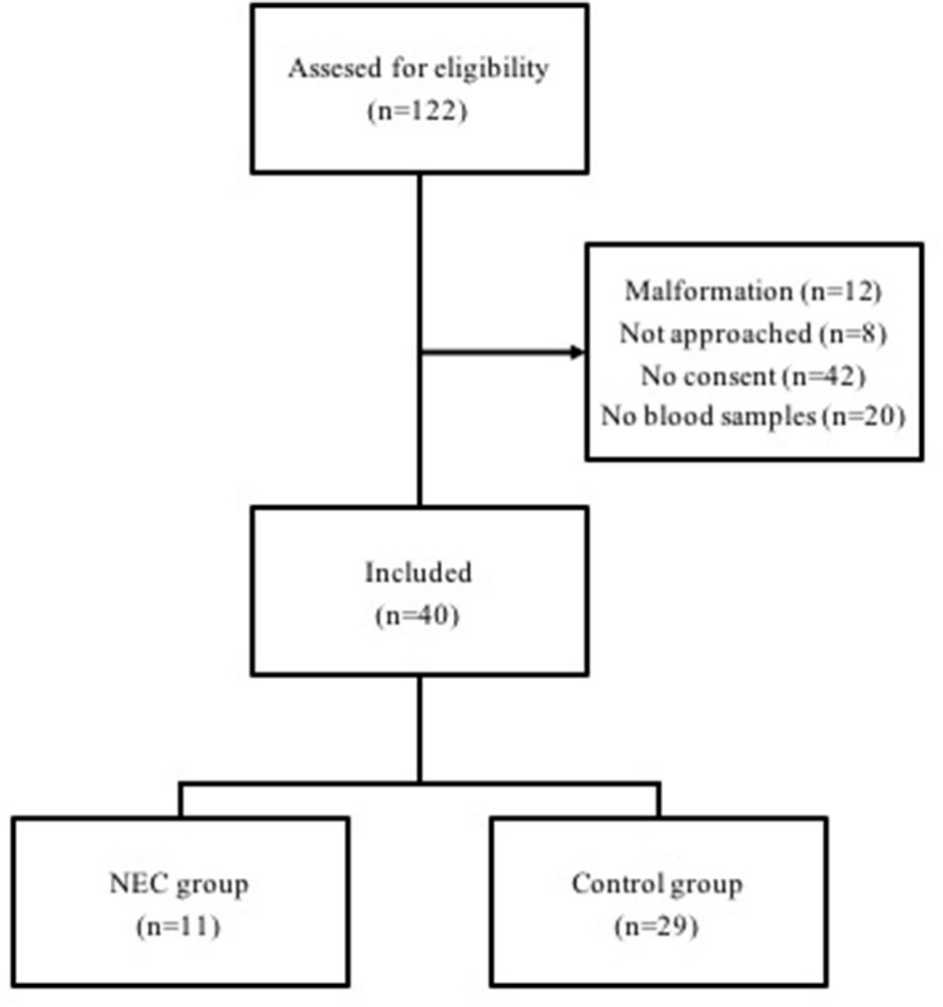
Flowchart of included infants (*n* = 40) born between 22 and 27 weeks of gestational age, and reasons for exclusion.

Infants born in November 2012–May 2015 at Uppsala University Children's Hospital before 28 weeks of gestation were prospectively included. Those with major congenital anomalies or heart defects were excluded. The population has previously been studied in the DAPPR-cohort (*Ductus Arteriosus and Pulmonary circulation in PReterm infants*) (29) and have been approved for study by the Regional Ethical Review Board in Uppsala, D:nr 2011/046. Out of 122 infants born during this period, 40 completed the full blood sampling needed in this study to obtain a level of significance of 5% and a power of at least 0.80 in this multi-parametrical study (Figure 1). All infants were included after informed and written consent from the parents was obtained. Eventually, 11 developed NEC and 29 controls did not. Background data from SNQ for the 122 individuals born during the period were used to compare background data in the study population, to ensure a reliable representation of the study period.

### Data Extraction and Study Variables

For each individual, clinical and laboratory parameters were studied. This included diagnosis of NEC defined as Bell stage ≥ IIa (19), as well as other associated diseases, such as PDA, intraventricular hemorrhage (IVH), bronchopulmonary dysplasia (BPD), respiratory distress syndrome (RDS), ROP, and infection/septicemia. Clinical features also included administration of prenatal steroids, preeclampsia, chorioamnionitis, delivery mode, twin birth, APGAR score, GA, BW, respiratory illnes, onset of illness, and mortality.

### Proximity Extension Assay (PEA)

Blood samples were collected from the umbilical arterial catheter on day 2 (median 1.7; range 1.5–2.0) after birth. The samples were centrifuged for 7 min at 2,400 × *g*, after which the serum was extracted to be stored at −80°C while awaiting analysis. A total of 202 biochemical markers (Table 1) were quantified in each individual with proximity extension assay (PEA), Olink, with the Proseek Multiplex 96 × 96 CVD I, Oncology I, and Inflammation I biomarker panels. PEA is suitable for serum analysis of pre-terms since it requires small blood volumes (1 μl) (30). Thirteen biomarkers were excluded due to analytical error (marked in Table 1). The final number of biomarkers in this study was 189.

**Table 1:** All quantified biomarkers (*n* = 202^*^).

4E-BP1	CCL28	CXCL10	ESM-1	hGDNF	IL-1ra	LEP	MMP-7	PTPN22	TM	VE-statin
ADA	CCL4	CXCL11	EZR	HGF	IL-2	LIF	MPO	PTX3	TNF	VIM
AGRP	CD244	CXCL13	FABP4	hK11	IL-20	LIF-R	MYD88	RAGE	TNFB	
AM	CD40	CXCL16	FADD	HSP 27	IL-20RA	LITAF	NEMO	REG-4	TNF-R1	
AR	CD40-L	CXCL5	FAS	ICOSLG	IL-22 RA1	LOX-1	NRTN	REN	TNF-R2	
ARTN	CD5	CXCL6	FasL	IFN-gamma	IL-24	LYN	NT-3	RETN	TNFRSF4	
AXIN1	CD6	CXCL9	FGF-19	IL-1 alpha	IL27-A	mAmP	NT-pro-BNP	SCF	TNFRSF9	
BAFF	CD69	Dkk-1	FGF-21	IL-10	IL-2RB	MB	NTRK3	SELE	TNFSF14	
BDNF	CDCP1	DNER	FGF-23	IL-10RA	IL-33	MCP-1	OPG	SIRT2	t-PA	
Beta-NGF	CDH3	ECP	FGF-5	IL-10RB	IL-4	MCP-2	OSM	SLAMF1	TRAIL	
BNP	CDKN1A	EGF	Flt3L	IL-12	IL-5	MCP-3	PAPPA	SPON1	TRAIL-R2	
CA-125	CEA	EGFR	FR-alpha	IL-12B	IL-6	MCP-4	PAR-1	SRC	TRANCE	
CAIX	CHI3L1	eIF-4B	FS	IL-13	IL-6RA	MIA	PARK7	ST1A1	TR-AP	
CASP-3	CSF-1	EMMPRIN	FUR	IL-15RA	IL-7	MIC-A	PDGF subunit B	ST2	TSLP	
CASP-8	CST5	EN-RAGE	GAL	IL-16	IL-8	MIP-1 alpha	PD-L1	STAMPB	TWEAK	
CCL11	CSTB	Ep-CAM	Gal-3	IL-17A	ILT-3	MK	PECAM-1	TF	uPA	
CCL19	CTSD	EPO	GDF-15	IL-17C	ITGA1	MMP-1	PlGF	TGF-alpha	U-PAR	
CCL20	CTSL1	ErbB2/HER2	GH	IL-17RB	ITGB1BP2	MMP-10	PRL	THPO	VEGF-A	
CCL23	CX3CL1	ErbB3/HER3	HB-EGF	IL-18	KLK6	MMP-12	PRSS8	TIE2	VEGF-D	
CCL25	CXCL1	ErbB4/HER4	HE4	IL-18R1	LAP TGF-beta-1	MMP-3	PSGL-1	TIM	VEGFR-2	

* *The 13 biomarkers excluded due to analytical error are shaded*.

### Clustering and Identification of Control Groups

Automatic cluster analysis of biomarker levels was performed with Cluster 3.0 (31), in which all biomarker levels were weighed equally. The two-dimensioned hierarchical multivariate analysis outlines the Euclidean distance between two factors or groups. Results from the cluster analysis were displayed as a map of color pixels with Java Treeview (32).

The controls were divided automatically into three subgroups by the clustering program (Control 1, Control 2, and Control 3) according to their biomarker expression patterns. After this, biomarker levels were statistically compared between the control groups, to ensure statistical support for the identified groups. New clustering was performed to explore how the NEC group clusters with the controls. A multivariate logistic regression to verify correlation found in the cluster analysis was not possible due to the amount of variables.

### Statistical Analysis

Statistical analysis was performed in Excel Version 15.27 (161010) and SPSS (1.0.0. 1447 64-bit edition). A *p*-value was considered statistically significant when <0.05. All tests of significance were two-tailed. The expression level of each biomarker was compared with a Student's *t*-test. After this, a Benjamini–Hochberg analysis was performed to reduce the risk for false-positive results. The q-value for this explorative study was set at 0.1. Background data within the study population (*n* = 40) were compared in the NEC group (*n* = 11) and the control group (*n* = 29) with Student's *t*-test for parametric data and the Mann–Whitney U for non-parametric data. Pearson's correlation was used to determine whether a biomarker level correlated to GA or BW. Background data from SNQ for the individuals born during the period (*n* = 122) were compared with data from the study population (*n* = 40) by chi-square test for nonparametric values and Student's *t*-test for parametric values.

## Results

No statistical difference was found in biomarker expression levels (*n* = 189) when comparing the NEC group with all controls: however, heterogeneities in controls could be further studied (see in Appendix 1).

### Identification of Control Groups Through Cluster Analysis

**Figure 2:**
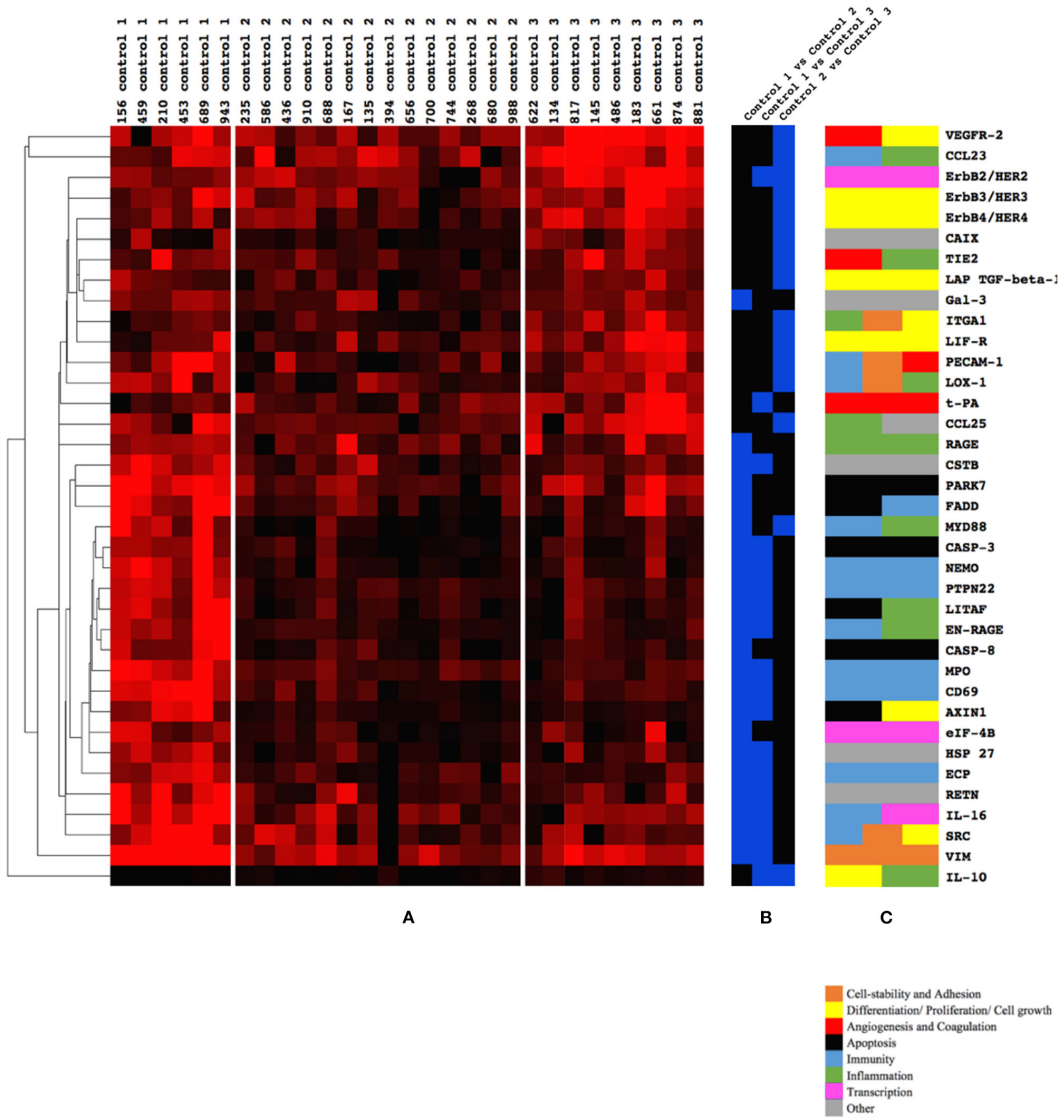
**(A–C)** Biomarkers (*n* = 37) that differed between the three Control groups, 1, 2, and 3 (blue), and their physiological function (brown, yellow, red, black, light blue, green, pink, and gray). **(A)** Three control subgroups could be identified by clustering of all biomarkers (*n* = 189). Significantly different biomarkers (*n* = 37) between the three control groups (1, 2, and 3) are presented. Higher intensity of red color indicates a higher expression of a given biomarker. **(B)** The comparison in which the biomarker differed in a comparison. Blue color indicates a significant difference of expression between control groups listed at the top of the column. Note that 23 biomarkers differed between Controls 1 and 2; 16 between Controls 1 and 3; and only 14 between Controls 2 and 3. **(C)** The function/functions of each biomarker as defined by www.humanproteinatlas.org (brown, yellow, red, black, light blue, green, pink, and gray).

Two-dimensional hierarchical clustering of biomarkers was performed including only controls to explore subgroups with unique biomarker profiles. The clustering software identified three control groups: Control 1, Control 2, and Control 3. Each control group had a unique pattern of biomarker expression. After statistical analysis, 37 biomarkers were identified to differentiate between any combinations of the control groups (Figure 2). Thus, each control group was characterized by an individual biomarker expression pattern. The expression pattern of the control groups, the differences in the expression levels between the groups, and the biological function of the presented biomarkers are shown in Figure 2. In Appendix 1, all biomarkers (*n* = 189) are clustered in all individuals (*n* = 40), where most of the NEC patients form a cluster with infants from Control 3.

### Biomarkers of Significance

**Figure 3:**
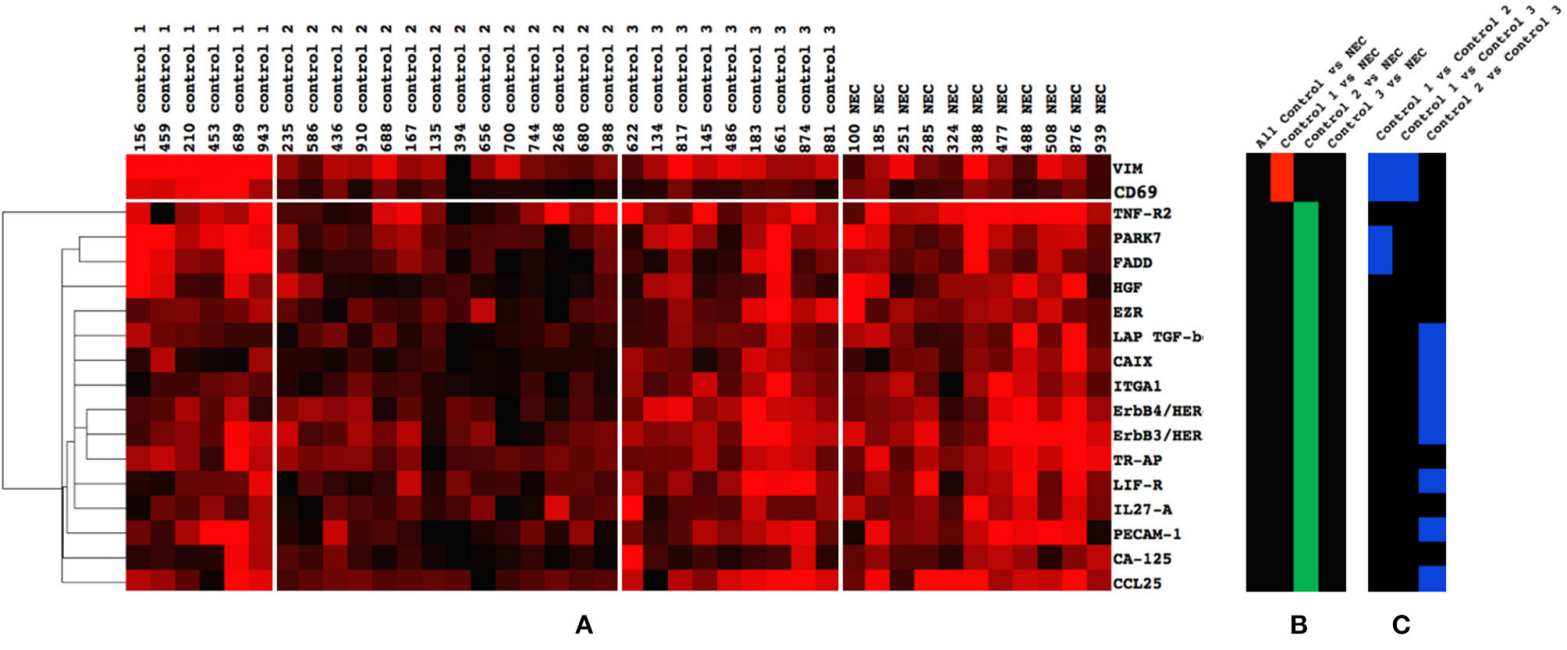
**(A–C)** Biomarkers (*n* = 18) that differed between Controls 1, 2, and 3 and the necrotizing enterocolitis (NEC) group. **(A)** Cluster of biomarkers (*n* = 18) that exhibited significant difference in expression when the NEC group was compared with all controls or separate control groups (1, 2, and 3). **(B)** Significant difference between controls and NEC. A red color is an indication that the mean expression level of the given protein was lower in NEC patients as compared with the control group. A green color signifies that the mean expression in the NEC patients was higher than in the control group. Note the higher expression of two biomarkers in Control 1 compared with the NEC group (*red*) and the lower expression of 16 biomarkers in Control 2 compared with the NEC group (green). Note also that no biomarker differed between all controls and the NEC group (black), nor between Control 3 and the NEC group (black). **(C)** Comparison of the 18 NEC specific biomarkers between Controls 1, 2, and 3. A blue color indicates a difference in expression between the two control groups listed at the top of the column. Note that four biomarkers differed between Controls 1 and 2 (blue), two biomarkers between Controls 1 and 3 (blue), and eight biomarkers between Controls 2 and 3 (blue). Only two biomarkers differed between all three control groups (*VIM* and *CD69*). The biological function according to www.humanproteinatlas.org of the six factors TNF-R2, HGF, EZR, TR-AP, IL-27A, and CCL25 are as follows: TNF-R2, apoptosis; HGF, differentiation/proliferation/cell growth; EZR, cell stability and adhesion; TR-AP, other (blood pressure regulation); IL-27A, immunity; and CCL25, inflammation and immunity.

Eighteen biomarkers differed when comparing the NEC group with any combination of the three control groups (Figure 3). Two biomarkers differed when comparing NEC with Control 1, six biomarkers differed when comparing the NEC group with Control 2, and no biomarker (*n* = 189) differed in expression when comparing the NEC group with Control 3. This seems to be visually confirmed when all controls were clustered together with NEC patients, as most of the NEC patients appeared in the same cluster as Control 3 (Appendix 2). The direction (+/–) of differences in biomarker expression between NEC patients and control groups are displayed in Figure 3B. Figure 3C shows the differences of the given biomarker between the control groups.

### Clinical Characteristics of the Study Population

Prenatal steroids were administered to the all individuals (*n* = 40). Incidence of the comorbidities RDS, IVH, BPD, infection, ROP, and persistent pulmonary hypertension of the newborn (PPHN) was compared in *NEC group vs. all controls* as well as *NEC group vs. control groups 1, 2, and 3*; however, no difference in incidence was found (Appendix 3). Twin pregnancy, preeclampsia, chorioamnionitis, and APGAR score (at 1, 5, and 10 min) did not differ between the NEC group, all controls, and Control groups 1, 2, and 3 (Appendix 3). A cluster analysis of clinical parameters along with significant biomarkers is displayed in Appendix 2.

The ratio of NEC in the individuals born during the study period (*n* = 122) was 16.4%, similar to that of the study group (*n* = 40, 27.5%, *p* = 0.211). The mortality during the 2 years was 25.4%, and in the study population, 17.5% (*p* = 0.412). The controls in the study population (*n* = 29) who did decease (*n* = 3) lived at least 3 months.

**Figure 4:**
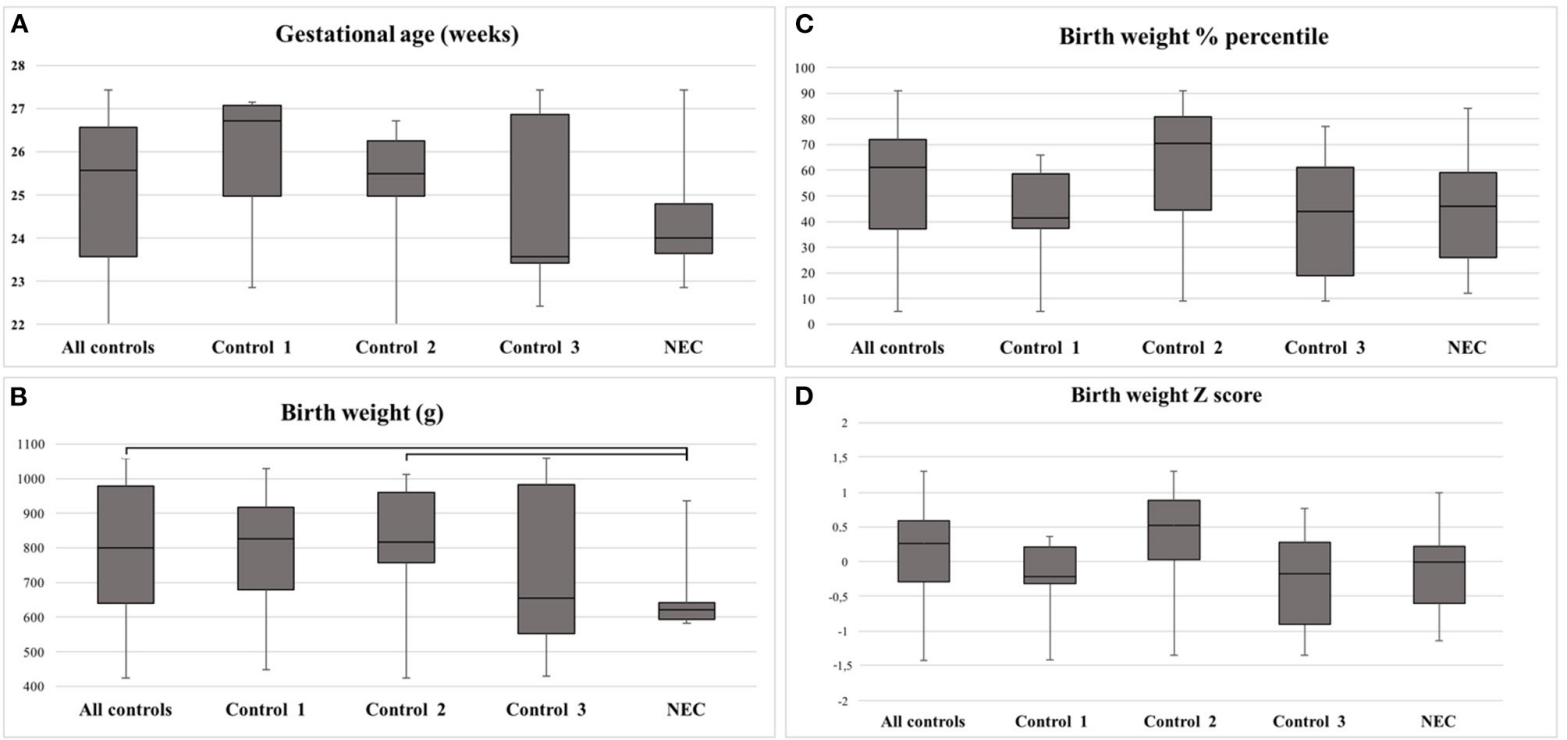
**(A–D)** Clinical data for the study population and detected differences. **(A)** Gestational age (GA) in weeks and days. **(B)** Birth weight (BW) in grams. **(C)** BW in percentiles. **(D)** BW in Z score. Note that BW differed between all controls and the necrotizing enterocolitis (NEC) group, as well as between Control 2 and the NEC group. Bars depict significant differences. Statistical calculations displayed in Table 2.

Median GA of all individuals was 25 weeks (range 22–27). No difference was found in GA between the groups (Figure 4A; Table 2). The BW was lower in NEC as compared with all controls (*p* = 0.023), or with Control 2 (*p* = 0.026) (Figure 4B; Table 2). Differences disappeared when the BW was adjusted to BW percentile (BW%) and BW Z score (Figures 4C,D; Table 2). None of the infants were growth restricted, as BW Z score was >-2 SD (Figure 4D). Visually, there was a tendency toward lower median GA, BW%, and BW Z score in Control 3 as compared with the NEC group (Figures 4A,C,D); however, this could not be statistically confirmed (Table 2; Control 3 vs. NEC). The median GA of Control 1 appears to be higher than the rest of the study population (Figure 4A).

**Table 2:** Detected statistical differences in parameters presented in Figures 4A–D.

***p*** **-value**	**GA**	**BW**	**BW%**	**BW Z score**
All controls vs. NEC	0.116	**0.023**	0.451	0.434
Control 1 vs. NEC	0.122	0.209	0.817	0.713
Control 2 vs. NEC	0.139	**0.026**	0.139	0.075
Control 3 vs. NEC	0.630	0.407	0.912	0.085
Control 1 vs. Control 2	0.502	0.973	0.151	0.085
Control 1 vs. Control 3	0.318	0.680	0.910	0.953
Control 2 vs. Control 3	0.567	0.503	0.165	0.071

*GA, BW, BW%, and BW Z score were compared between NEC, all controls, and Control groups 1–3. Detected differences by clustering were analyzed by two-sided paired Student's t-test and corrected with Benjamini–Hochberg for multiple comparisons. A p-value of < 0.05 (presented in bold) was considered significant*.

*GA, gestational age; BW, birth weight; NEC, necrotizing enterocolitis*.

The median time from birth until diagnosis of NEC was 9 days (range 2–18). The mean GA of those developing NEC earlier than 9 days was 24.4 weeks, which was not higher (p = 0.882) than the mean GA of those who developed NEC later (mean GA 23.8).

### Correlation Analysis

A negative correlation was found between BW and expression of hepatocyte growth factor (HGF), ErbB3/HER3, and Erb4/HER4. For the rest of the biomarkers, no correlation between biomarker expression and BW or GA could be confirmed (Table 3).

**Table 3:** Correlations between biomarkers (*n* = 18) and GA or BW.

	**GA**		**BW**	
	***Pearson's coefficient***	***p*** **-value**	***Pearson coefficient***	***p*** **-value**
VIM	0.203	0.216	0.101	0.539
CD69	0.177	0.266	0.065	0.713
TNF-R2	0.080	0.623	0.165	0.294
PARK7	0.090	0.566	−0.082	0.610
FADD	0.069	0.668	−0.084	0.623
HGF	−0.291	0.059	**−0.500**	**0.001**
TR-AP	−0.235	0.153	−0.311	**0.051**
EZR	−0.090	0.566	−0.242	0.136
LAP TGF-beta-1	−0.042	0.806	−0.202	0.216
CAIX	−0.088	0.566	−0.213	0.566
ITGA1	−0.087	0.566	−0.146	0.356
ErbB3/HER3	−0.270	0.092	**−0.359**	**0.022**
ErbB4/HER4	**−0.402**	**0.011**	**−0.480**	**0.001**
TR-AP	−0.235	0.153	−0.311	**0.052**
LIF-R	−0.127	0.424	−0.104	0.540
IL27-A	**0.337**	**0.031**	0.224	0.173
PECAM-1	−0.061	0.713	−0.072	0.668
CA-125	−0.139	0.389	−0.167	0.278
CCL25	−0.202	0.216	−0.225	0.153

*Each significantly different biomarker (n = 18) between Controls 1 and 2 and NEC was analyzed with Pearson's correlation to see if the biomarker level correlated to gestational age or birth weight. Significant correlations (p < 0.05; presented in bold) were found between GA and ErbB4/HER4 or IL27-A (blue); BW and HGF, ErbB3/HER3, or ErbB4/HER4 (gray), but with a relatively low correlation coefficient of ≤ 0.50*.

*GA, gestational age; BW, birth weight; NEC, necrotizing enterocolitis*.

### Selected Analysis of Biomarkers Previously Studied in Necrotizing Enterocolitis and Colitis

**Figure 5:**
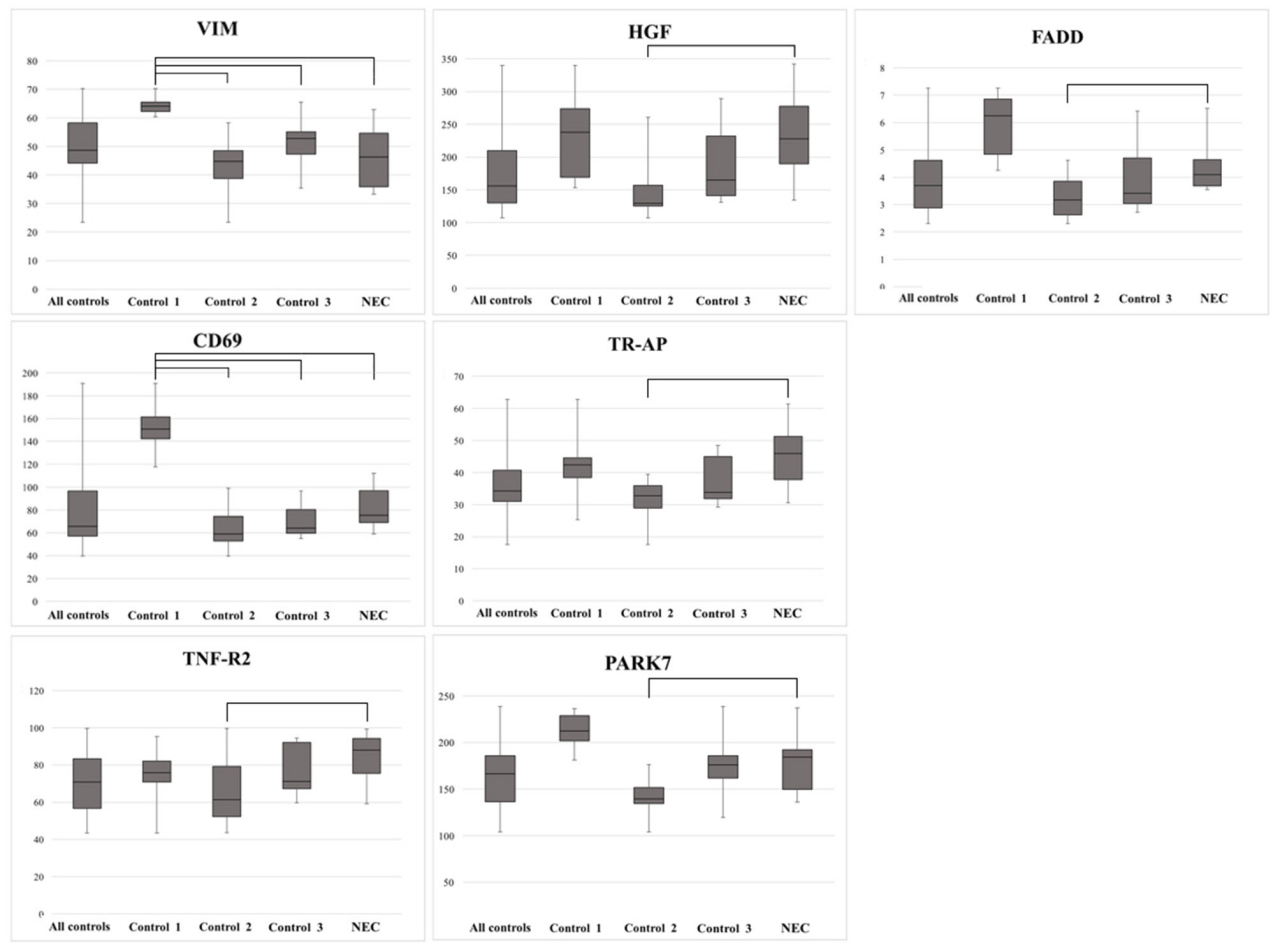
Biomarkers previously reported linked to necrotizing enterocolitis (NEC) (*n* = 7), and with the significant difference between Controls 1, 2, and 3 and the NEC group. Note the higher expression of VIM and CD69 in Control 1 compared with the NEC group, and the lower expression of TNF-R2, PARK7, FADD, HGF, and TR-AP in Control 2 compared with the NEC group. Bars depict significant differences.

A literature search in PubMed of the 18 biomarkers that differed between the control groups and the NEC group (Figure 2) showed that TNF-R2, HGF, and tartrate-resistant acid phosphatase 5 (TR-AP) have previously been described in relation to NEC. Fas-associated protein with death domain (FADD) and PARK7 have been reported in relation to colitis. Besides the expression of these five proteins, Figure 5 includes vimentin (VIM) and CD69, which were elevated in Control 1 as compared with all other groups. The levels of FADD, TNF-R2, HGF, TR-AP, and PARK7 were elevated in NEC compared with Control 2 (Figure 5).

## Discussion

In this study of 40 extremely preterm infants, 189 biomarkers with functions mostly in inflammation, proliferation, and vascularization were quantified at day 2 after birth in an effort to find potential early risks of emerging NEC. Eleven of 40 infants later developed NEC.

Our main finding is that no biomarker (*n* = 189) differed in expression when comparing infants who later developed NEC with all controls. Furthermore, all infants (*n* = 40) had comparable clinical perinatal history. This suggests that on day 2, the individuals in this high-risk group overall exhibit the same starting point in regard to inflammation, vascularization, and possibly in the risk of NEC development. This opens up for postnatal factors influencing which individuals go on to develop NEC and that prophylactic treatment and close monitoring very well can be beneficial in reducing NEC incidence.

The subdivision of the controls may be important to investigate variation of NEC risk shortly after birth. If a blood test taken at day 2 reveals biomarker patterns incongruent with those found in NEC, this could be an indication of lower risk of developing NEC. Contrariwise, a group of individuals exhibiting similar patterns as the NEC group could be suspected to have an increased risk of developing NEC. The latter group could benefit from close observation and prophylactic treatment.

Control 3 stands out as particularly interesting to compare with the NEC group. Besides similar clinical and perinatal parameters, not a single biomarker (*n* = 189) differed in expression when comparing Control 3 with those who later developed NEC. Control 3 even exhibited a visual trend toward lower median GA and adjusted BW than the NEC group. Both factors would essentially make Control 3 more prone to develop NEC (15) than the NEC group itself. Our data suggest that at day 2 after birth, Control 3 may exhibit the same risk of developing NEC as the individuals that later did develop NEC. Based on these observations, it seems feasible to hypothesize that postnatal, rather than perinatal, factors determine which individuals are at the highest risk of developing NEC in this cohort of extremely preterm infants.

Several biomarkers (*n* = 18) differed when comparing the NEC group with Control 1 and 2. Two of them were higher in Control 1 as compared with NEC, Control 2, and Control 3, namely, VIM and CD69.

VIM, a type III intermediate filament, is a component in the cytoskeleton (33). In rat models with inflammatory bowel disease (IBD), VIM expression was increased (34). Knock-out of VIM in mouse models with induced IBD has considerably less inflammation that in those with VIM (35). In our study, VIM is lower in NEC patients than Control 1, which is incongruent with the function of VIM.

CD69 expression indicates leucocyte activation and is an early marker of inflammation (36). CD69 has been found to be upregulated after intestinal bacterial exposure (37) and downregulated in murine models with severe anemia (38). Furthermore, CD69 is thought to reduce tissue damage from ischemia, by reducing endothelial activation (39) and has increased expression in blood cells after intake of probiotics in healthy adults (40). It also plays a role in immunosuppressive regulatory cells, through promotion of IL-10 production (41). We found lower levels of CD69 in NEC patients compared with Control 1, which is not conflicting in with the functions described above.

In a risk evaluation, we observed that a simultaneously higher expression of VIM and CD69 on day 2 indicated a lower risk for NEC. There was a visual tendency toward higher GA in Control 1, which could affect biomarker levels. However, in the correlation analysis of VIM and CD69 with clinical data, expression levels could not be linked to GA and BW.

The homogeneity in clinical characteristics of the patients both with and without NEC signifies a suitable basis for analysis, since it minimizes the risk of confounding factors influencing biomarker concentrations. The inverse correlations between NEC and GA and/or BW are well-known major risk factors for NEC (15). In the present study, we found only a tendency for such a correlation, probably because the study population consisted of extremely preterm infants, thus focusing on individuals already at the highest risk of developing NEC.

Sixteen biomarkers were lower in Control 2 as compared with the NEC group. Twelve of these also differed in expression when comparing the three control groups, while six biomarkers (Ca-125, IL27A, TR-AP, EZR, HGF, and TNF-R2) did not. Since these six biomarkers do not differ when comparing NEC with Control 3, they could be what indicates a high risk of developing NEC. Some of these proteins have previously been linked to colitis.

We found that TNF-R2 was lower in a group of controls, which is in accordance with previous NEC studies (42, 43). Tumor necrosis factor has pleiotropic effects with both pro- and anti-inflammatory effects (44). TNF-R2 has been postulated to be a pro-inflammatory mediator in the pathophysiology of NEC (42). Increased TNF-R2 signaling in mice has been found to induce intestine barrier loss, resulting in colitis (45, 46). TPN nutrition further contributes to TNF dysregulation of the epithelial barrier function in mouse models (45). The increased level of TNF-R2 found in the NEC group could be an indication of a predisposed compromised intestinal barrier.

HGF regulates cell proliferation, cell survival, and angiogenesis (47), which are especially important in enterocytes (48). In our study, we found that HGF expression was inversely correlated to BW and a tendency toward it being inversely correlated to GA. Inverse correlation of GA and HGF expression has been found previously (49) and would be in line with the fact that increased prematurity increases risk for NEC (15). Protein levels are higher in the second trimester as compared with levels found in urine from newborns (50). Although HGF correlation to BW in preterm has not been fully studied, HGF has been described to be a biomarker for being SGA (51). Being SGA is a risk factor for NEC (15). In contrast, it has been found that fetal swallowing of amniotic fluid containing HGF decreases NEC incidence in rats (52) and that induced colitis yields greater damage in HGF-deficient mice (53). To summarize, high HGF could be an indication of increased immaturity and being SGA.

TR-AP is a serum marker for activated macrophages and chronic inflammation and is being explored for diagnosis of chronic inflammatory diseases (54). TR-AP-positive macrophages reside in the lamina propria of the healthy colon, and a histopathological increase of TR-AP expression has been found in colitis-induced rats (55). NEC has to our knowledge not previously been linked to TR-AP. The elevated TR-AP expression in the NEC group could signify increased inflammatory activity.

Leukemia inhibitory factor receptor (LIF-R), carbonic anhydrase IX (CAIX), integrin alpha (ITGA), and their potential links to NEC and colitis have, to our knowledge, not been reported. Although no link has previously been made between FADD and NEC, FADD has been found to prevent intestinal inflammation (56), and knock-out of FADD has been shown to induce colitis (57). Parkinson's disease protein 7 (PARK7) deficiency leads to increased apoptosis in colitis and has been proposed as a therapeutic target for colitis (58) but has never been linked to NEC.

There are previous studies on the link between NEC/colitis and expression of ezrin (EZR) (59, 60), tumor growth factor beta 1 (TGF-β1) (42), VIM (34, 35), IL-10 (61), epidermal growth factor receptor 3 (ErbB3/HER3) (62), epidermal growth factor receptor 4 (ErbB4/HER4) (63, 64), platelet endothelial cell adhesion molecule 1 (PECAM-1) (65, 66), carcinoma antigen 125 (CA-125) (67, 68), and chemokine ligand 25 (CCL25) (69, 70). These findings are not congruent with the direction of expression level (+/–) in NEC patients compared with controls in this study. However, this analysis is not a study of biomarker level in stated NEC but an attempt to determine levels before onset of fulminant NEC. To this date, few data are available for such comparison.

### Strengths and Limitations

The reason for not finding differences in biomarkers between NEC and all controls could be that day 2 after birth is too early to detect relevant biomarkers. Given the single blood sampling, it was not possible to consider the chance of biomarker levels being influenced by diurnal variation, or whether day 2 after birth is the optimal time to find valuable biomarkers. Further studies with sequential blood sampling would be beneficial to take these factors into account.

A strength of this study is its benchmarking of biomarker patterns along with perinatal clinical data to explore risk of NEC development. The results from this study can be used for comparison in future studies. The single, early blood sampling illustrates the starting point of extremely preterm infants before being influenced by postnatal factors. While we hypothesize that postnatal factors influence why Control group 3 did not develop NEC, it was not possible to verify this in this study due to the data not being collected.

The reason for the relatively high NEC incidence in the study group (27%) as compared with national data on extremely preterm infants (9%) (71) could be the fact that survival until day 2 was necessary to be included in the study, since this was the time of blood sampling. This excluded infants who died from early causes of death, such as asphyxia, respiratory conditions, IVH, congenital anomalies, and early infections (72–75). The relatively high incidence of NEC could also be ascribed to the low GA in the entire study group.

## Conclusion

In this study of extremely preterm infants, the expression of early comprehensive biomarkers (*n* = 189) at day 2 of life could not distinguish those who later developed NEC from all controls. Thus, the study could not identify biomarkers that can be used to select infants at high risk of developing NEC when comparing the NEC group with all controls. After subdivision of controls into three groups, simultaneously elevated VIM and CD69, or simultaneously lower expression of TNF-R2, PARK7, FADD, HGF, and TR-AP, could be regarded as a lower risk for developing NEC in some of the infants. Known risk factors of NEC were not higher in individuals who later developed NEC, which suggests that postnatal factors influence NEC development.

## Data Availability Statement

The raw data supporting the conclusions of this article will be made available by the authors, without undue reservation.

## Ethics Statement

The studies involving human participants were reviewed and approved by Etikprövningsmyndigheten, Uppsala, Sweden. Written informed consent to participate in this study was provided by the participants' legal guardian/next of kin. Written informed consent was obtained from the individual(s), and minor(s)' legal guardian/next of kin, for the publication of any potentially identifiable images or data included in this article.

## Author Contributions

AH performed collection of data, statistical analysis, wrote and edited the manuscript. LM performed data collection analysis, statistical analysis as well as writing and revising of the manuscript. HL partook in data analysis and in writing and revising the manuscript. KO set up the study, performed data collection, statistical analysis, and revised the manuscript. RS set up the study, performed data collection, data analysis, and writing and revising of the manuscript. The manuscript has been read and approved for submission by all authors. All authors approve this version to be published.

## Funding

This study was funded by H.K.H. Kronprinsessan Lovisas Förening för Barnasjukvård (Grant Number 2018-00459).

## Conflict of Interest

The authors declare that the research was conducted in the absence of any commercial or financial relationships that could be construed as a potential conflict of interest.

## Publisher's Note

All claims expressed in this article are solely those of the authors and do not necessarily represent those of their affiliated organizations, or those of the publisher, the editors and the reviewers. Any product that may be evaluated in this article, or claim that may be made by its manufacturer, is not guaranteed or endorsed by the publisher.
