# Supplementary material for: Early Postnatal Comprehensive Biomarkers Cannot Identify Extremely Preterm Infants at Risk of Developing Necrotizing Enterocolitis
Source: Front Pediatr. 2021 Oct 22;9:755437. doi: 10.3389/fped.2021.755437 (PMC8570110; doi:10.3389/fped.2021.755437)
Supplement: Supplementary file 1 [file Data_Sheet_1.PDF]

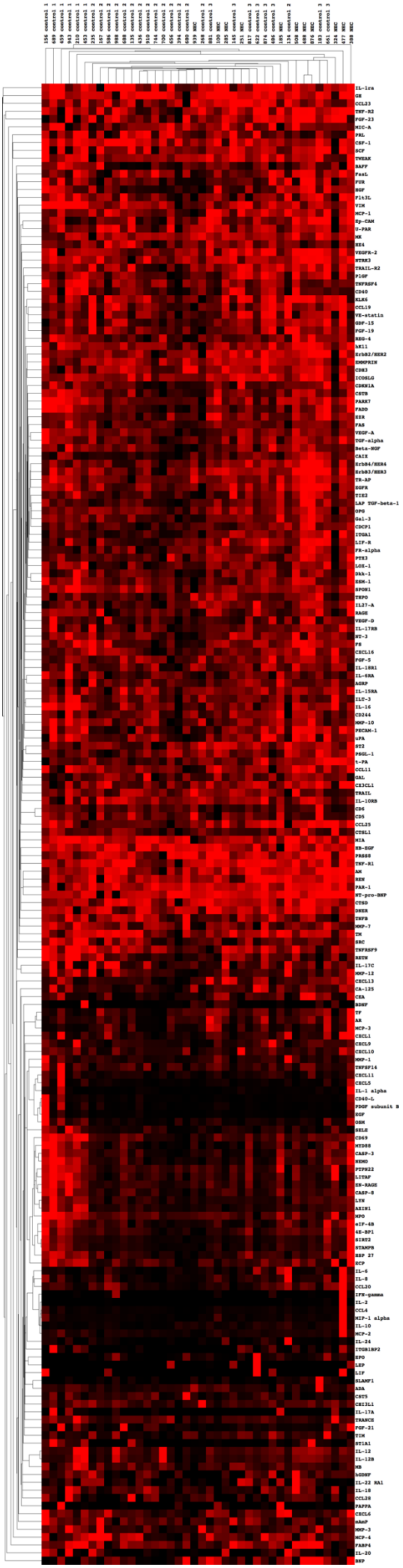

Appendix 1. All biomarkers (n=189) clustered in all infants (n=40). Note the automatic clustering of controls into 3 subgroups (Controls 1-3 in upper part of the figure), and how (besides infant No. 939) the infants with NEC are mixed with Control 3.

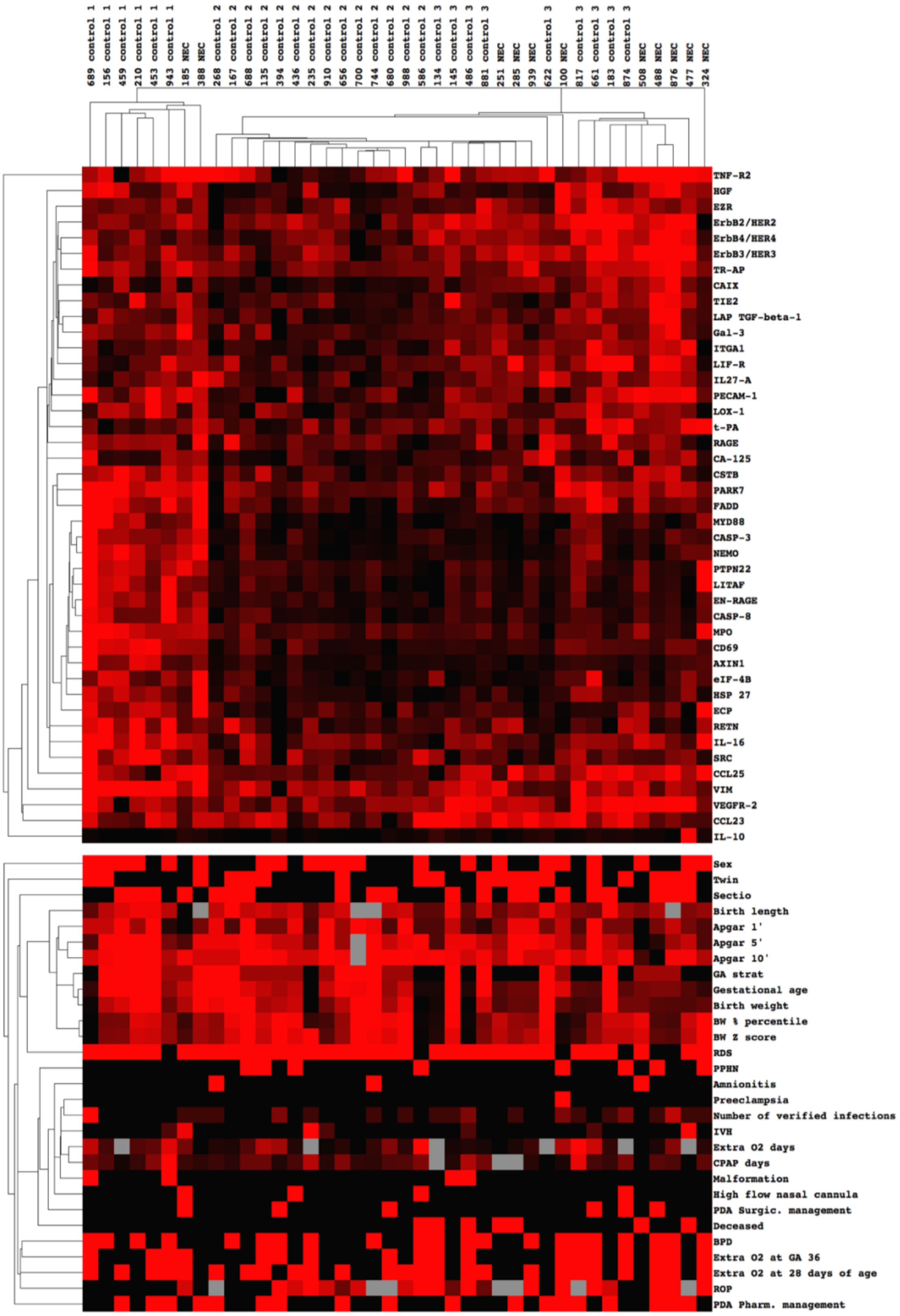

Appendix 2. Clustering of significant biomarkers (n=43) and clinical features. The individuals that later developed NEC are clustered mainly with control group 3.

Appendix 3.

**Clinical characteristics of the Study Population**					
	All Controls<br>N=29	Control 1<br>N=6	Control 2<br>N=14	Control 3<br>N=9	NEC<br>N=11
Birth weight, grams – median (range)	800 (423-1058)	826 (448-1028)	816 (423-1012)	654 (468-1058)	621 (582-935)
Gestational age, weeks – median (range)	25 (22-27)	26 (22-26)	25 (22-27)	23 (22-27)	24 (22-27)
Male sex – no (% in group)	15 (52)	2 (33)	7 (50)	6 (67)	2 (18)
RDS – no (% in group)	26 (90)	5 (83)	13 (93)	8 (89)	8 (73)
IVH – no (% in group)	5 (17)	1 (17)	2 (14)	2 (22)	2 (18)
BPD – no (% in group)	16 (55)	4 (67)	6 (43)	6 (67)	5 (45)
Late infection – no (% in group)	12 (41)	1 (17)	5 (36)	6 (67)	8 (73)
ROP – no (% in group)	14 (48)	0 (9)	8 (36)	6 (67)	6 (55)
PPHN – no (% in group)	5 (17)	0 (0)	4 (29)	1 (11)	3 (27)
Pre-eclampsia – no (% in group)	0 (0)	0 (0)	0 (0)	0 (0)	1 (9)
Twin – no (% in group)	12 (41)	2 (33)	5 (36)	5 (56)	7 (64)
Chorioamnionitis - no (% in group)	2 (7)	0 (0)	2 (14)	0 (0)	1 (9)
Antenatal steroids - no (% in group)	29 (100)	6 (100)	14 (100)	9 (100)	11 (100)
Apgar 1' – median (range)	5 (2-9)	6 (2-8)	6 (3-9)	4 (2-9)	4 (1-7)
Apgar 5' – median (range)	7 (3-9)	8,5 (3-9)	7 (4-9)	6 (3-9)	5 (1-9)
Apgar 10' - median (range)	8 (6-10)	8,5 (6-10)	8 (7-10)	8 (7-10)	7 (1-10)

RDS, respiratory distress syndrome; IVH, intraventricular haemorrhage; BPD, bronchopulmonary dysplasia; ROP, retinopathy of prematurity; PPHN, persistent pulmonary hypertension of the neonate
